# Metabolic analysis of radioresistant medulloblastoma stem-like clones and potential therapeutic targets

**DOI:** 10.1371/journal.pone.0176162

**Published:** 2017-04-20

**Authors:** Lue Sun, Takashi Moritake, Kazuya Ito, Yoshitaka Matsumoto, Hironobu Yasui, Hidehiko Nakagawa, Aki Hirayama, Osamu Inanami, Koji Tsuboi

**Affiliations:** 1 Department of Radiological Health Science, Institute of Industrial Ecological Sciences, University of Occupational and Environmental Health, Japan, Kitakyushu, Fukuoka, Japan; 2 Department of Radiobiology, Graduate School of Comprehensive Human Sciences, University of Tsukuba, Tsukuba, Ibaraki, Japan; 3 Proton Medical Research Center, Faculty of Medicine, University of Tsukuba, Tsukuba, Ibaraki, Japan; 4 Central Institute of Isotope Science, Hokkaido University, Sapporo, Hokkaido, Japan; 5 Laboratory of Organic and Medicinal Chemistry, Graduate School of Pharmaceutical Sciences, Nagoya City University, Nagoya, Aichi, Japan; 6 Center for Integrative Medicine, Tsukuba University of Technology, Tsukuba, Ibaraki, Japan; 7 Laboratory of Radiation Biology, Department of Applied Veterinary Sciences, Graduate School of Veterinary Medicine, Hokkaido University, Sapporo, Hokkaido, Japan; Instituto Nacional de Cardiologia, MEXICO

## Abstract

Medulloblastoma is a fatal brain tumor in children, primarily due to the presence of treatment-resistant medulloblastoma stem cells. The energy metabolic pathway is a potential target of cancer therapy because it is often different between cancer cells and normal cells. However, the metabolic properties of medulloblastoma stem cells, and whether specific metabolic pathways are essential for sustaining their stem cell-like phenotype and radioresistance, remain unclear. We have established radioresistant medulloblastoma stem-like clones (rMSLCs) by irradiation of the human medulloblastoma cell line ONS-76. Here, we assessed reactive oxygen species (ROS) production, mitochondria function, oxygen consumption rate (OCR), energy state, and metabolites of glycolysis and tricarboxylic acid cycle in rMSLCs and parental cells. rMSLCs showed higher lactate production and lower oxygen consumption rate than parental cells. Additionally, rMSLCs had low mitochondria mass, low endogenous ROS production, and existed in a low-energy state. Treatment with the metabolic modifier dichloroacetate (DCA) resulted in mitochondria dysfunction, glycolysis inhibition, elongated mitochondria morphology, and increased ROS production. DCA also increased radiosensitivity by suppression of the DNA repair capacity through nuclear oxidization and accelerated the generation of acetyl CoA to compensate for the lack of ATP. Moreover, treatment with DCA decreased cancer stem cell-like characters (e.g., CD133 positivity and sphere-forming ability) in rMSLCs. Together, our findings provide insights into the specific metabolism of rMSLCs and illuminate potential metabolic targets that might be exploited for therapeutic benefit in medulloblastoma.

## Introduction

Brain tumors are the leading cause of cancer-related death in children, responsible for 7 per 10^6^ deaths in the USA and approximately 10 per 10^6^ deaths in Japan; medulloblastoma is the most common malignant pediatric brain tumor, accounting for 20% of pediatric brain tumors in the USA and 12% in Japan [1–4]. Although overall survival rates for medulloblastoma patients have improved in recent years the morbidity rate remains significant, with survivors often suffering from adverse neurologic, endocrinologic, and social effects with the current treatment options [5–10]. Consequently, there is an urgent need to better understand the mechanism of therapy refractoriness and to develop novel and specific tumor therapies with reduced brain toxicity for medulloblastoma patients.

Recent molecular-based classifications divide medulloblastomas into four subtypes to allow more accurate patient stratification and an appropriate clinical approach for each patient [9, 11]. However, it has been shown that medulloblastoma is composed of heterogeneous cancer cell populations due to cell differentiation within individual tumors, including tumor cells with stem cell-like properties termed medulloblastoma cancer stem-like cells (CSLCs) together with other cancer cells [12, 13]. Previous clinical and biological evidence indicates that CSLCs have tumor reconstruction capacity and are more resistant to radiation and conventional chemotherapy than non-CSLCs, suggesting an important role in tumor recurrence [14–17]. Understanding medulloblastoma CSLCs in more depth will aid development of efficient and effective novel therapies for medulloblastoma.

The energy metabolic pathway is largely differentiated between cancer and normal cells. In particular, cancer cells exhibit higher glycolytic activity than normal cells and increased ^18^fluoro-2-deoxyglucose (FDG) avidity on positron emission tomography (PET). Glycolytic ATP generation is crucial for cancer cells because glycolysis bifurcates into anabolic pathways producing essential nucleotides, lipids, and amino acids for proliferation [18]. Interestingly, recent studies have reported that pluripotent stem cell metabolism shifts from oxidative phosphorylation to aerobic glycolysis, similar to that observed in most cancers [19, 20]. During differentiation, pluripotent stem cells downregulate glycolysis and switch to utilizing glycolysis-derived pyruvate in their mitochondria through oxidative phosphorylation [21]. It is obvious that energy metabolic pathways and mitochondria are important to maintain stem cell-like phenotypes in normal cells and, as a corollary, we might assume that energy metabolic pathways and mitochondrial function are also important for CSLCs to maintain their stem cell-like phenotype.

Several research groups have reported the metabolic preference of CSLCs in glioma, breast cancer, pancreatic cancer, and leukemia [22–25]; however, the results are controversial. For example, Feng et al. showed that a population enriched for breast tumor-initiating cells relies more heavily on glycolysis than non-tumorigenic cancer cells, with decreased pyruvate dehydrogenase (Pdh) expression [22]. However, Lagadinou et al. showed that leukemia stem cells are metabolically dormant and dependent on oxidative respiration rather than glycolysis for energy generation, and rely on BCL-2–mediated oxidative respiration for energy homeostasis [23]. The metabolic properties and specific metabolic pathways that sustain stem cell-like phenotypes and radioresistance of medulloblastoma CSLCs remain areas of active investigation.

We previously established radioresistant medulloblastoma stem cell-like clones (rMSLCs), ONS-F8, ONS-B11, and ONS-F11, by irradiation of the ONS-76 human medulloblastoma cell line [26]. These established clones show a high expression level of CD133, tumor sphere-forming ability, side population ratio, and radioresistance compared with parental ONS-76 cells [26]. Additionally, rMSLCs can be stably maintained in the same culture conditions as parental ONS-76 cells and readily conform to experimental conditions. The first aim of this study was to identify the characteristics of metabolic pathways in medulloblastoma CSLCs in our experimental system using established rMSLCs and parental ONS-76 cells.

Dichloroacetate (DCA) is a small molecule that promotes Pdh activity by inhibition of pyruvate dehydrogenase kinase (Pdk). Originally, DCA was developed for the treatment of lactic acidosis, but recent research indicates that DCA is also a prospective drug for several diseases associated with metabolic change, including cancer and heart failure [27–29]. In particular, previous reports have demonstrated that administration of DCA suppresses tumor growth, increases cancer cell radiosensitivity, and reduces glycolysis-dependent CSLC maintenance [22, 27, 30, 31]. The potential mechanism for this DCA-induced anticancer effect is through promotion of oxidative phosphorylation and reactive oxygen species (ROS) generation, which in turn stimulates apoptosis, autophagy, and cell cycle arrest [27, 30, 32–34]. However, it is largely unknown how such changes occur in central carbon metabolism, including glycolysis and the tricarboxylic acid (TCA) cycle, following DCA treatment, and how these metabolic changes cause increased ROS production and extinction of stem cell-like properties and radioresistance in medulloblastoma CSLCs. Thus, the second purpose of this study was to examine these phenomena in rMSLCs after administration of DCA.

The results of this study revealed that rMSLCs displayed lower oxidative phosphorylation activity and higher pyruvate kinase (PYK) activity and lactate production than parental cells. Moreover, intervention with DCA modified multiple steps of glycolysis and the TCA cycle and resulted in increased cellular oxidative stress and altered mitochondria morphology, thus suppressing cancer stem cell-like phenotypes and radioresistance. Our findings also indicate that CSLC metabolism plays an important role in maintaining cancer stem cell-like phenotypes and radioresistance, and provide greater insight into the development of metabolic targeting radiotherapy to disrupt medulloblastoma CSLCs.

## Materials and methods

### Cell line and culture conditions

The human ONS-76 medulloblastoma cell line was obtained from the RIKEN Cell Bank (Tsukuba, Ibaraki, Japan). ONS-F8 and ONS-B11 cell lines were established from ONS-76 after irradiation as previously described [26]. The cells were cultured in minimal essential medium (MEM; Sigma-Aldrich Inc., Tokyo, Japan) containing 10% fetal bovine serum (FBS; Nichirei Biosciences Inc., Tokyo, Japan), 100 mg/ml streptomycin, and 100 U/ml penicillin (Sigma-Aldrich). Cells were incubated in a humidified atmosphere at 37°C with a 5% CO_2_ atmosphere. For subcultures, cells were rinsed with Ca^2+^- and Mg^2+^-free PBS (Sigma-Aldrich), and dispersed with 0.25% trypsin containing 0.5 mM ethylenediaminetetraacetate (EDTA; Sigma-Aldrich). A final concentration of 50 mM DCA (Sigma-Aldrich) was added 48 h before analysis. The number of cells was determined with TC10^™^ (Bio-Rad, Tokyo, Japan).

### Tumorigenicity assay in immune-deficient mice

Cells were trypsinized and cell densities ranging from 100 to 1×10^6^ cells were suspended in 200 μl of a 1:1 mixture of MEM and Matrigel^™^ (Becton Dickinson). Cells were transplanted subcutaneously into NOD/SCID mice (4- to 6-week-old males; CLEA Japan, Inc., Tokyo, Japan). Tumors were monitored 16 weeks after transplantation [35].

### Intercellular ROS analysis

Intercellular ROS levels were detected by incubation with 10 μM 5-(and-6)-chloromethyl-2′,7′-dichlorodihydrofluorescein diacetate, acetyl ester (CM-H2DCFDA; Life Technologies, Tokyo, Japan) in culture media for 30 min. Cells were trypsinized and resuspended in MEM, and at least 3×10^4^ cells were analyzed by flow cytometry (FACSCalibur; Becton Dickinson, Franklin Lakes, NJ, USA). The mean fluorescence intensity of each sample was normalized to that of the ONS-76 control.

### Measurement of nuclear redox state

Nuclear-specific oxidative stress was measured using a bisbenzimide-nitroxides probe that localizes to cell nuclei and shows a substantial increase in fluorescence upon exposure to ROS [36]. Cells were incubated with 10 μM bisbenzimide-nitroxides probe in culture media for 3 h, trypsinized, and suspended in PBS. At least 1×10^4^ cells were analyzed by flow cytometry (MoFlo XDP; Beckman Coulter Inc., Brea, CA, USA). The mean fluorescence intensity of each sample was normalized to that of ONS-76.

### Analysis of mitochondria mass, membrane potential, and superoxide production

Mitochondria mass, mitochondria membrane potential, and mitochondria-derived superoxide were detected using the fluorescent probes MitoGreen (2 μM; PromoKine, Heidelberg, Germany), MitoRed (2 μM; PromoKine), and MitoSOX Red (5 μM; Life Technologies), respectively. The cells were incubated with MitoGreen and MitoRed for 30 min and with MitoSOX for 10 min in culture media, trypsinized, and suspended in MEM. At least 3×10^4^ cells were analyzed by flow cytometry (FACSCalibur). The mean fluorescence intensity of each sample was normalized to that of ONS-76.

### Measurement of oxygen consumption rate (OCR) by MitoXpress

OCR was measured using a MitoXpress Xtra-Oxygen Consumption Assay kit (Luxcel Biosciences, Cork, Ireland), according to the recommended protocol. Briefly, cells were cultured in 96-well plates (CulturPlate F; PerkinElmer Japan, Kanagawa, Japan) and the culture medium was replaced with fresh medium before experiments. Oligomycin (1 μM; Sigma-Aldrich) or carbonyl cyanide 4-(trifluoromethoxy)phenylhydrazone (FCCP; 1 μM; Wako Pure Chemical Industries, Osaka, Japan) were added to each well, followed by 10 μl of reconstituted MitoXpress reagent and two drops of HS Mineral Oil. The plate was immediately measured kinetically in a fluorescence plate reader (Varioskan LUX; Thermo Fisher Scientific, Kanagawa, Japan) at 380/650 nm excitation/emission every 20 s over a period of 30 min.

### Measurement of oxygen consumption rate (OCR) by electron spin resonance (ESR)

The oxygen probe 5,9,14,18,23,27,32,36-octa-n-butoxy-2,3-naphthalocyanine (LiNc-BuO) has been described previously [37, 38]. Cells were trypsinized, washed in MEM, and 1.25×10^5^ cells were suspended with 0.2 mg LiNc-BuO and 2% dextran before being drawn into a glass capillary tube. The tube was sealed at both ends and subjected to X-band electron spin resonance spectroscopy (FA200; JEOL, Tokyo, Japan) every 2 min. The cavity was maintained at 37°C using a temperature controller (ES-DVT4; JEOL). ESR conditions were as follows: microwave frequency 9.4466 GHz, microwave power 1 mW, center field 322.650 mT, sweep width 0.5 mT, sweep time 1 min, and time constant 0.1 s. The spectral line widths were analyzed using a WinRad (Radical Research, Tokyo, Japan). Line width was converted into O_2_ values and OCR was calculated as described previously [37, 38].

### Fluorescence microscopy of mitochondria

Cells were fixed with 4% paraformaldehyde (PFA; Wako Pure Chemical Industries) at room temperature for 10 min and washed three times in PBS before incubation with 2 μM MitoGreen in PBS at room temperature for 30 min in the dark. Cells were washed three times and mounted with fluorescence mounting medium (DAKO, Carpinteria, CA, USA). Images were acquired with an inverted fluorescence microscope (BZ-X700, Keyence, Tokyo, Japan).

### Transmission electron microscopy (TEM)

Cells were trypsinized and fixed with 2.5% glutaraldehyde in PBS for 12 h at 4°C and washed three times with PBS. Cells were post-fixed in 1% osmium tetroxide for 2 h at 4°C and washed three times with PBS. Cells were sequentially dehydrated using 50%, 70%, 80%, 95%, and 100% ethanol (10 min each), incubated in Epon solution (3 times, 1 h each), and maintained at 37°C for 12 h, 45°C for 12 h, and 60°C for 2 days. Ultra-thin sections (70 nm) were made using an ultra-microtome and imaged using a JEM-1400 transmission electron microscope (JEOL). At least 50 mitochondria in each sample were analyzed by ImageJ software (NIH, USA). Mitochondria circularity was calculated from the equation C = 4*πS*/*L*^2^, where S is the mitochondria area and L is the mitochondria perimeter.

### Measurement of CD133-positive ratio

Cells were trypsinized, washed with PBS containing 2% FBS, and 2×10^6^ cells were suspended in 80 μl of PBS with 2% FBS prior to addition of 20 μl FcR blocking reagent (Miltenyi Biotec Inc., Tokyo, Japan) and 10 μl of phycoerythrin (PE)-conjugated anti-human CD133/1 mouse IgG1 antibody (Miltenyi Biotec). After incubation for 30 min at 4°C in the dark cells were washed twice with 2% FBS in PBS and propidium iodide (PI) (Sigma-Aldrich) was added to a final concentration of 1 μg/ml to detect dead cells. The cells were filtered through a 35-μm cell strainer and at least 3×10^4^ cells were analyzed by flow cytometry (FACSCalibur).

### Tumor sphere assay

Cells were cultured in serum-free medium (SFM) composed of Dulbecco’s Modified Eagle Medium/Nutrient Mixture F-12 (DMEM/ F12; GIBCO, Life Technologies), 20 ng/ml epidermal growth factor (EGF; (Sigma-Aldrich), 20 ng/ml basic fibroblast growth factor (bFGF; Sigma-Aldrich) and 20 μl/ml B27 supplement (GIBCO, Life Technologies). Cells were plated at a density of 1,000 cells/well on ultra-low attachment surface 24-well plates (Corning Inc., Lowell, MA, USA). DCA was added to treatment groups to a final concentration of 50 mM. The number of spheres was counted at day 10.

### Clonogenic assay

Cells in flasks were exposed to X-irradiation (130 kVp, 5 mA, approximately 0.9 Gy/min) at doses of 2, 4, 6, and 8 Gy. After irradiation, the cells were trypsinized and counted, and the predetermined number of cells was plated onto three 60-mm dishes (Falcon, Becton Dickinson) for each dose point. After incubation for 14 days, the colonies were fixed and stained with 0.25% methylene blue solution (Wako) in 90% ethanol solution. The number of surviving colonies that included 50 cells or more was counted and averaged [39]. Survival curves were fitted to the linear-quadratic model using DeltaGraph v.5.4 software (RedRock Software, Inc., Salt Lake City, UT, USA) as previously described [40]. Sensitivity enhancement ratio at 4 Gy (SER _(4.0)_) was calculated from the equation SER _(4.0)_ = log Sf _(DCA, 4.0)_ / log Sf _(4.0)_, where Sf _(DCA, 4.0)_ and Sf _(4.0)_ were surviving fractions at 4 Gy with and without DCA administration, respectively.

### DNA agarose gel electrophoresis

After X-ray irradiation with 20 Gy, the cells were trypsinized and embedded into 0.6% megabase-agarose gel plugs. The plugs were treated with proteinase K in lysis buffer for 1 h on ice, incubated at 50°C overnight, treated with 0.1 mg/ml RNase in TE buffer at 37°C for 1 h, and embedded into a 0.6% megabase-agarose gel. DNA was fractionated by electrophoresis in Tris-borate-EDTA running buffer (Wako) and visualized with an ImageQuant LAS 4000 (GE healthcare Japan, Tokyo, Japan).

### Metabolite analysis

Metabolite analysis was performed using C-Scope (Human Metabolome Technologies, Yamagata, Japan), according to the recommended protocol [41]. Briefly, the cells were washed twice with 5% mannitol solution and treated with 0.8 ml of methanol and 0.55 ml of 8 μM internal standard (IS). After centrifugation at 2,300 g, 4°C for 5 min, the supernatants were collected for centrifugal filtration through a 5-kDa-cutoff filter at 9,100 g, 4°C for 3 h. The extracted metabolites were stored at −80°C until analysis. Concentrations of all charged compounds were measured by capillary electrophoresis time-of-flight mass spectrometry (CE-TOFMS) and capillary electrophoresis tandem mass spectrometry (CE-QqQMS; CE-MS/MS) as described previously [41, 42].

## Immunocytochemical staining of γ-H2AX

Cells were irradiated with 2 or 4 Gy and fixed in 4% PFA for 10 min at 0.5, 3, 6, and 24 h time points. The fixed cells were washed in PBS, permeabilized in 0.5% Triton X- 100 (Wako), rinsed again in PBS, and blocked with 10% donkey serum (Wako) in PBS with 0.5% Tween-20 (Wako) for 60 min at room temperature. The cells were sequentially incubated for 1.5 h with a 1:1,000 dilution of human monoclonal anti-phospho-histone H2AX (Ser139) antibody (EMD Millipore, Darmstadt, Germany) in PBS with 1% donkey serum buffer and for 1 h with a 1:1,000 dilution of Alexa Fluor^®^ 488-conjugated donkey anti-mouse IgG secondary antibody (Life Technologies) in blocking buffer. The cells were counterstained with 4',6-diamidino-2-phenylindole (DAPI; Wako), mounted on slide glasses with DAKO fluorescent mounting medium (Dako), and viewed using a fluorescence microscope (BZ-X700, Keyence).

### Measurement of extracellular lactate production

Extracellular lactate production was measured using the Lactate Assay Kit (BioVision, California, USA), according to the recommended protocol. Briefly, cells were plated in 6-well plates and fresh medium containing 100 mM 2-deoxy-D-glucose (2DG; Sigma-Aldrich) was added 24 h before experimentation. Culture medium (1 μl/well) was added to 96-well plates and the volume was adjusted to 50 μl/well with Lactate Assay Buffer before addition of 50 μl Reaction Mix (containing 46 μl Lactate Assay Buffer, 1 μl Lactate Enzyme Mix, and 1 μl Probe) to each well and incubation at room temperature in the dark for 10 min. Absorbance (OD 570 nm) was measured using a MTP-300 microplate reader (Corona Electric, Ibaraki, Japan). Lactate concentration was derived from absorbance using a standard curve.

### Pyruvate kinase (PYK) activity

PYK activity was measured using the Pyruvate Kinase Assay Kit (Biovision), according to the recommended protocol. Briefly, cells were trypsinized and 3×10^5^ cells were suspended in PK Assay Buffer. The cells were disrupted by a Sonifier 250 (Emerson, Kanagawa, Japan) and centrifuged at 10,000 g, 4°C for 10 min. Cell extracts were added to 96-well plates and the volume was adjusted to 50 μl/well with PK Assay Buffer before addition of 50 μl Reaction Mix (containing 44 μl PK Assay Buffer, 2 μl PK Substrate Mix, 2 μl PK Enzyme Mix, and 2 μl OxiRed Probe) to each well and incubation at room temperature in the dark for 20 min. Absorbance (OD 570 nm) was measured using a MTP-300 microplate reader (Corona Electric). PYK activity concentration was derived from absorbance using a standard curve.

### Measurement of glucose uptake

Glucose uptake was measured using a Glucose Assay Kit (Abcam, Tokyo, Japan), according to the recommended protocol. Briefly, cells were plated in 6-well plates and fresh medium was added 24 h before experimentation. Culture medium (1 μl/well) was added to 96-well plates and the volume was adjusted to 50 μl/well with Lactate Assay Buffer before addition of 50 μl Glucose Reaction Mix (composed of 46 μl Glucose Assay Buffer, 1 μl Glucose Enzyme Mix, and 1 μl Glucose Probe) to each well and incubation at room temperature in the dark for 10 min. Absorbance (OD 570 nm) was measured using a Varioskan LUX microplate reader (Thermo Fisher Scientific). Glucose concentration was derived from absorbance using a standard curve. Glucose uptake was calculated from the equation: Glucose uptake = Glucose concentration in fresh medium—Glucose concentration in cell culture medium.

### Statistical analysis

The mean and standard deviation (SD) were calculated for each data point. Welch’s t test was used to analyze significant differences between groups. A P value less than 0.05 was considered statistically significant.

### Ethical considerations

All animal experiments were performed in accordance with the Animal Care Guidelines of the University of Tsukuba. All animal husbandry procedures and animal experiments were consistent with the University of Tsukuba’s Regulation of Animal Experiment and were approved by the Animal Experiment Committee, University of Tsukuba (Permit Number: 12–414 and 14–078). Mice were sacrificed when the tumor size reached 1.0 cm in diameter. Mice were sacrificed by administering CO_2_.

## Results

### rMSLCs had higher tumorigenic ability than parental ONS-76 cells

We previously established rMSLCs with high stem cell-like phenotypes, including high CD133 positivity, side population ratio, and sphere-forming ability, by γ-ray irradiation of ONS-76 cells (S1 Fig) [26]. In this study, we first determined the tumorigenicity of ONS-F8 and ONS-B11, the two clones with highest CD133 ratio, by transplanting them into NOD/SCID mice. ONS-F8 and -B11 readily generated tumors even after transplantation of only 100 cells, whereas at least 1,000 parental ONS-76 cells were required for tumor formation (S1 Fig). Tumorigenic cell frequencies calculated using the formula available on the WEHI ELDA website [43] for ONS-76, -F8, and -B11 were 1 in 4,747, 1 in 1,351 and 1 in 1,508, respectively (S1 Fig). These results showed that ONS-F8 and -B11 had higher tumorigenic ability than the parental ONS-76 cells.

### rMSLCs displayed low ROS levels associated with mitochondria superoxide production

**Fig 1:**
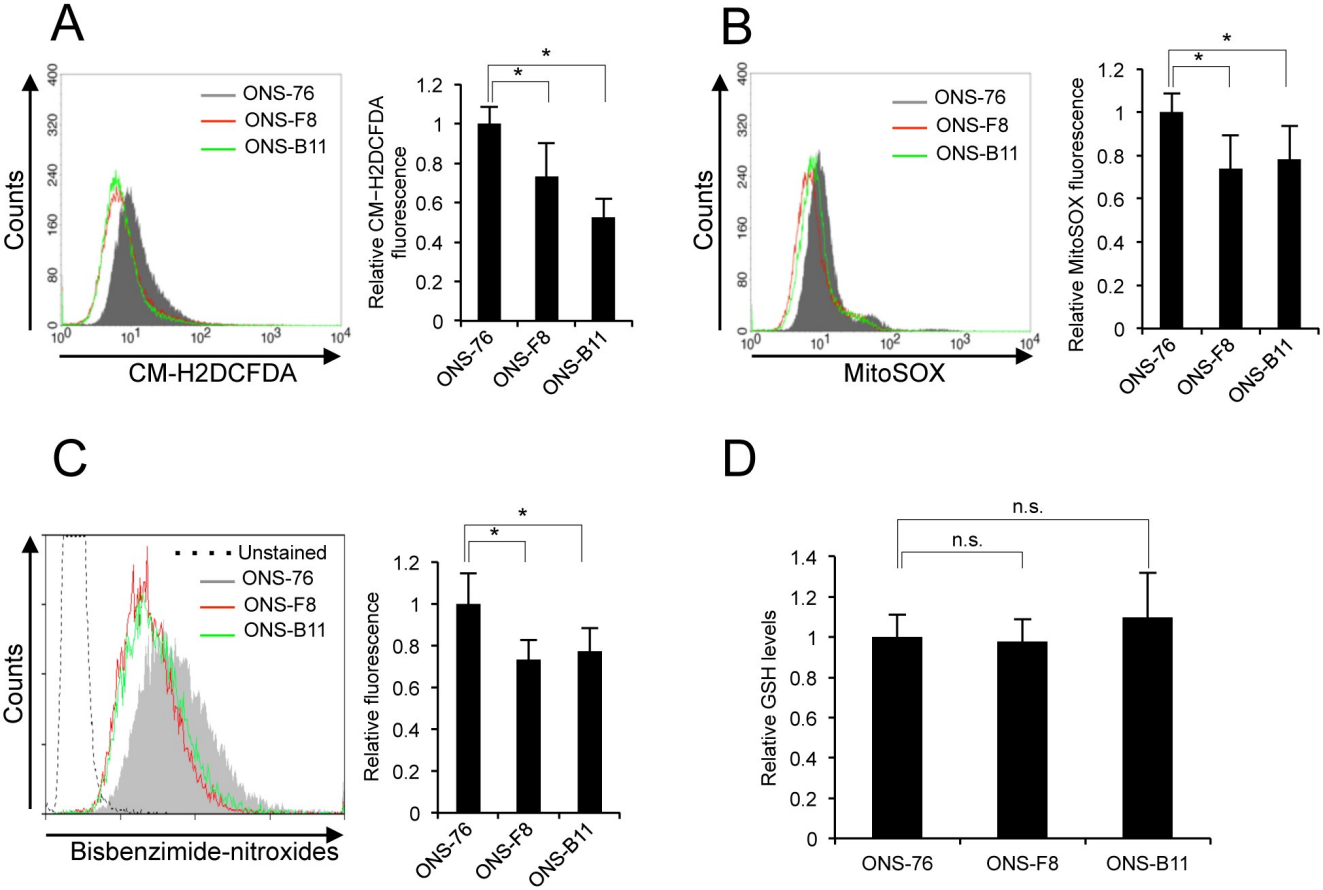
ONS-F8 and -B11 showed low levels of ROS and nuclear oxidative stress. Levels of (A) intracellular ROS, (B) mitochondria-derived superoxide, (C) nuclear oxidative stress, (D) GSH in ONS-76, -F8 and -B11. All quantitative data are means ± S.D. *P<0.05, Welch’s t-test, n.s., non-significant.

As low ROS levels are maintained in several types of normal stem cells and mitochondrial ROS generation is highly reliant on oxidative phosphorylation [44–46], we initially measured intracellular ROS levels, mitochondria superoxide production, and nuclear oxidative stress levels using the CM-H2DCFDA, MitoSOX, and bisbenzimide-nitroxides fluorescent probes, respectively. Both ONS-F8 and -B11 showed lower intracellular ROS concentration, mitochondria ROS generation, and nuclear oxidative stress levels than parental cells (Fig 1A–1C).

CSLCs show an enhanced capacity for synthesis of reduced GSH and defense against ROS [16, 47]. Given the low ROS levels in ONS-F8 and -B11, we investigated whether these cells had elevated levels of GSH. Contrary to our initial expectation, levels of cellular GSH were not significantly different among ONS-76, -F8, and -B11 (Fig 1D). These results suggest that low intracellular ROS levels and nuclear oxidative stress levels are preferentially maintained by low endogenous mitochondria superoxide production in ONS-F8 and -B11.

### Diminution of oxidative phosphorylation in rMSLCs

**Fig 2:**
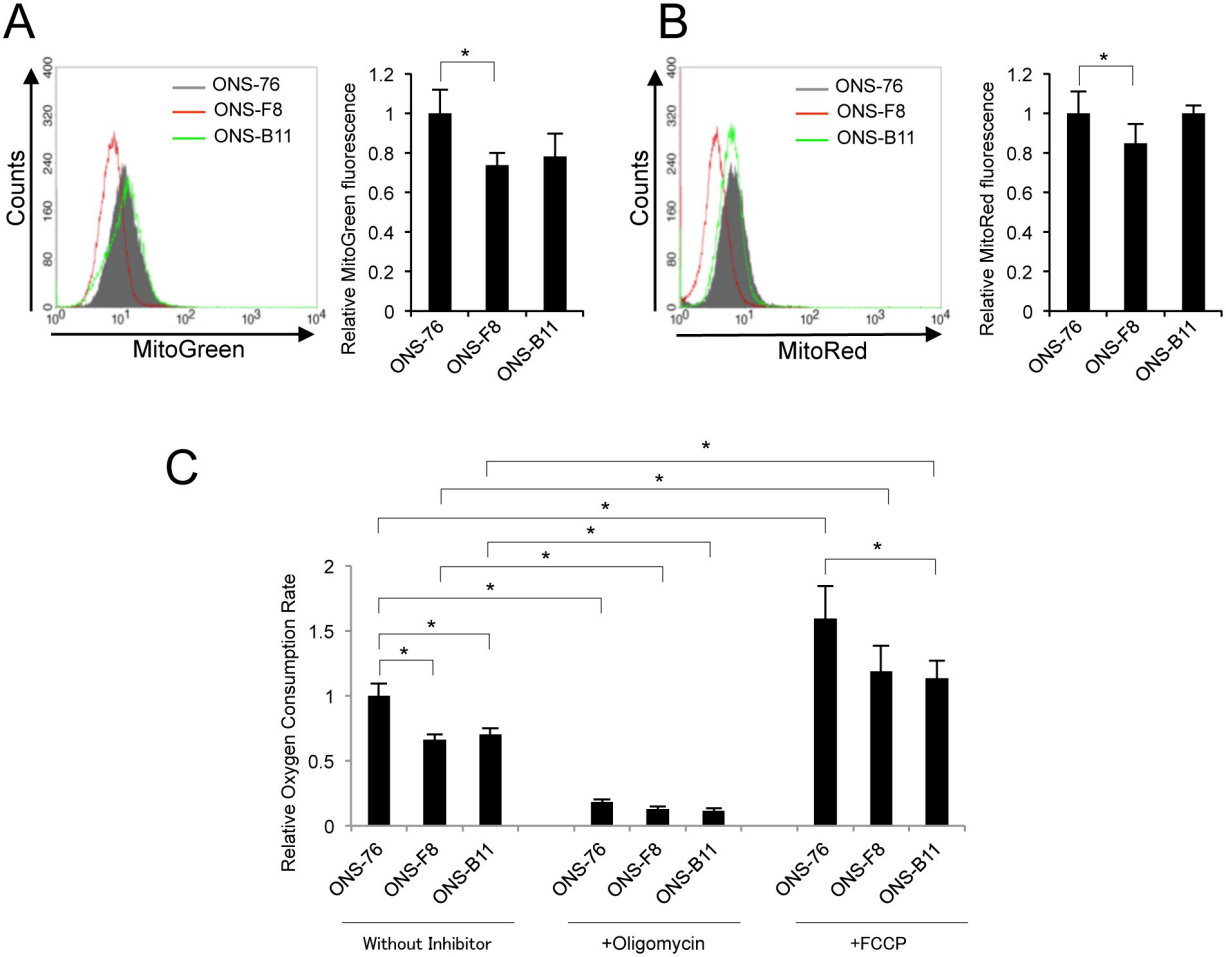
Diminution of mitochondrial respiration in ONS-F8 and -B11. (A) Mitochondria mass, (B) mitochondria membrane potential, (C) OCR (using MitoXpress) in ONS-76, -F8 and -B11 cells. All quantitative data are means ± S.D. *P<0.05, Welch’s t-test.

Given that electron leak from the electron transport chain is the primary cause of endogenous ROS generation [48, 49], we hypothesized that oxidative phosphorylation is reduced in rMSLCs. Measurement of mitochondria mass and membrane potential using MitoGreen and MitoRed respectively revealed that, compared to ONS-76 cells, ONS-F8 had lower mitochondria mass and membrane potential but ONS-B11 showed no significant difference (Fig 2A and 2B). To further substantiate the oxidative phosphorylation potential in ONS-76, -F8, and -B11 cells, we analyzed the oxygen consumption rate (OCR) using a MitoXpress OCR measurement kit. Both ONS-F8 and -B11 showed significantly lower basal OCR than ONS-76 (Fig 2C). The OCR was completely inhibited by oligomycin treatment, indicating that it reflects mitochondrial respiration (Fig 2C). These results suggest that ONS-F8 and -B11 had lower oxidative phosphorylation levels than parental cells. FCCP significantly increased OCR in ONS-76, -F8, and -B11. ONS-F8 and -B11 showed lower OCR than ONS-76 in the presence of FCCP (Fig 2C).

To support this observation, we further analyzed OCR by ESR spectrometry using LiNc-BuO as an oxygen-sensing probe [37]. The cells were mixed with LiNc-BuO particles in culture medium and analyzed by ESR [38] in a time-dependent manner; OCR was calculated from the change in LiNc-BuO spectrum line width. ONS-F8 and -B11 cells showed significantly lower basal OCR than ONS-76 (S2 Fig). These results were consistent with the data obtained by MitoXpress.

### DCA induces alteration of ROS metabolism and mitochondria function in rMSLCs

**Fig 3:**
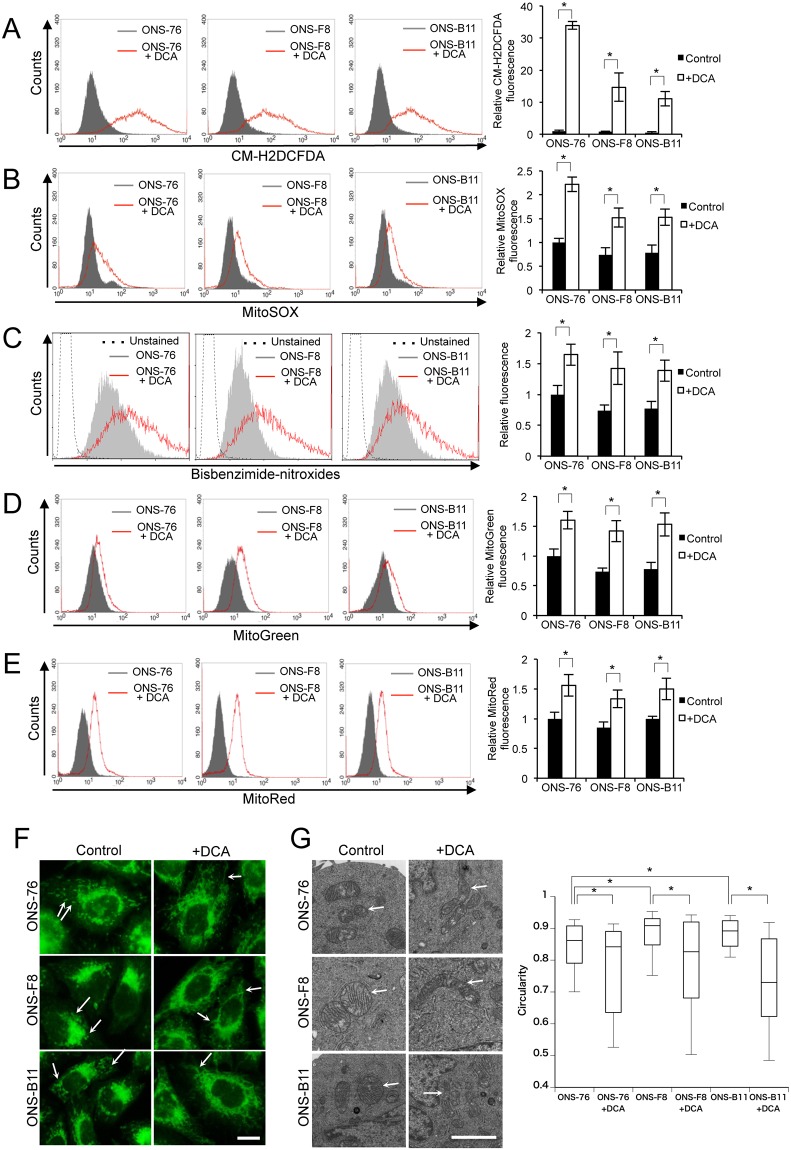
DCA enhanced ROS production and changed mitochondrial signature. (A) Intracellular ROS, (B) mitochondria-derived superoxide, (C) nuclear oxidative stress, (D) mitochondria mass, (E) mitochondria membrane potential, (F) fluorescence microscopy with MitoGreen stain (scale bar 5 μm), and (G) TEM analysis of mitochondria (scale bar 2 μm) and mitochondrial circularity (at least 50 mitochondria were measured for each sample using ImageJ software) in ONS-76, -F8 and -B11 cells with and without DCA. White arrows indicate mitochondria. Quantitative data (without mitochondrial circularity) are means ± S.D. Quantitative data of mitochondrial circularity are shown as boxplots. *P<0.05, Welch’s t-test.

To examine the relationship between ROS levels and mitochondria function in rMSLCs, we examined ROS levels and mitochondria function after DCA treatment. Following treatment with DCA (50 mM, 48 h), intracellular ROS concentration, mitochondria ROS generation, nuclear oxidative stress levels, mitochondria volume, and mitochondria membrane potential were significantly increased in ONS-76, -F8, and -B11 (Fig 3A–3E). Mitochondria morphology is related to mitochondria oxidative phosphorylation and ROS generation [50–52]. Immunofluorescent staining and TEM analysis showed that mitochondria in the DCA-treated cells were predominantly elongated and formed extensive reticular networks (Fig 3F and 3G).

We next determined the toxicity of DCA (50 mM, 48 h) using PI staining and the clonogenic assay. There were no significant differences in the PI-positive cell ratio and colony-forming efficiency between DCA-treated and control cells (S3 Fig). These results demonstrated that DCA treatment induced alterations in ROS metabolism and mitochondria function in rMSLCs without affecting cell survival under our experimental conditions.

### DCA induces alteration of mitochondrial respiration and glycolysis

**Fig 4:**
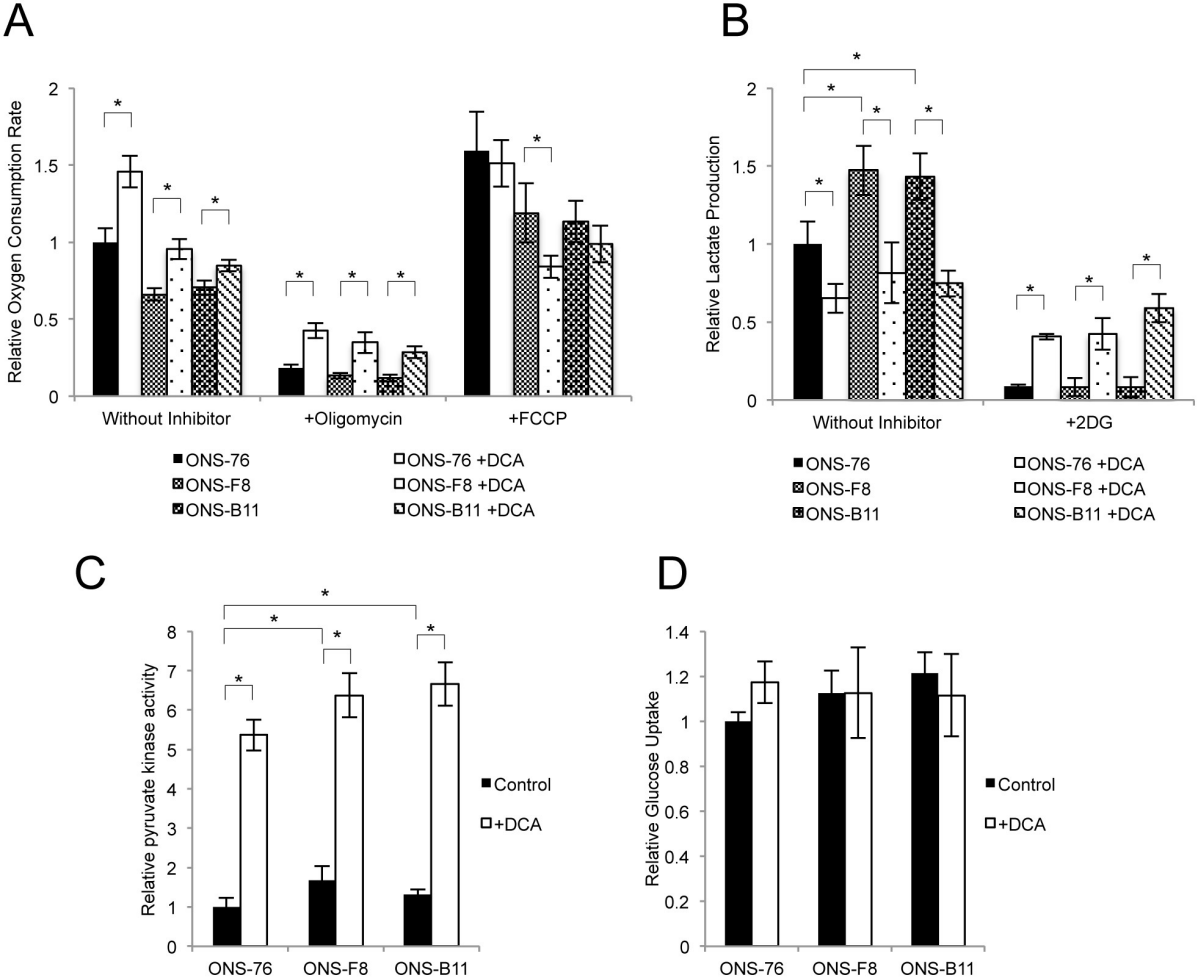
DCA induces alteration of mitochondrial respiration and glycolysis. (A) OCR (using MitoXpress), (B) extracellular lactate production, (C) pyruvate kinase activity, (D) glucose uptake in ONS-76, -F8, and -B11 cells with and without DCA. All quantitative data are means ± S.D. *P<0.05, Welch’s t-test.

To investigate the effect of DCA treatment on mitochondrial respiration and glycolysis we examined OCR, extracellular lactate production, the activity of PYK (a glycolysis enzyme that converts PEP to pyruvate while generating ATP), and glucose uptake in control and DCA-treated cells. DCA significantly increased OCR in ONS-76, -F8, and -B11 cells (Fig 4A). In the presence of oligomycin, DCA-treated cells showed significantly higher OCR than non-treated cells suggesting that the increased oxygen was not used for ATP production in oxidative phosphorylation. In the presence of FCCP, DCA-treated cells showed the same (ONS-76 and -B11) or lower (ONS-F8) OCR compared with non-treated cells (Fig 4A).

ONS-F8 and -B11 showed higher extracellular lactate production than ONS-76 cells, and DCA significantly decreased lactate production relative to untreated cells. Lactate production was completely inhibited by 2DG treatment in the absence of DCA, indicating that the lactate production reflects glycolysis (Fig 4B). Interestingly, in the presence of 2DG, DCA-treated cells showed significantly higher lactate production than untreated cells, suggesting that lactate was produced by another metabolic pathway (Fig 4B). ONS-F8 and -B11 showed high PYK activity and the same level of glucose uptake compared with ONS-76 (Fig 4C and 4D). DCA significantly increased PYK activity relative to untreated cells, but did not affect glucose uptake (Fig 4C and 4D). These results demonstrated that DCA treatment induced alterations in mitochondrial respiration and glycolysis in rMSLCs.

### rMSLCs lose cancer stem-like phenotypes *in vitro* after DCA treatment

**Fig 5:**
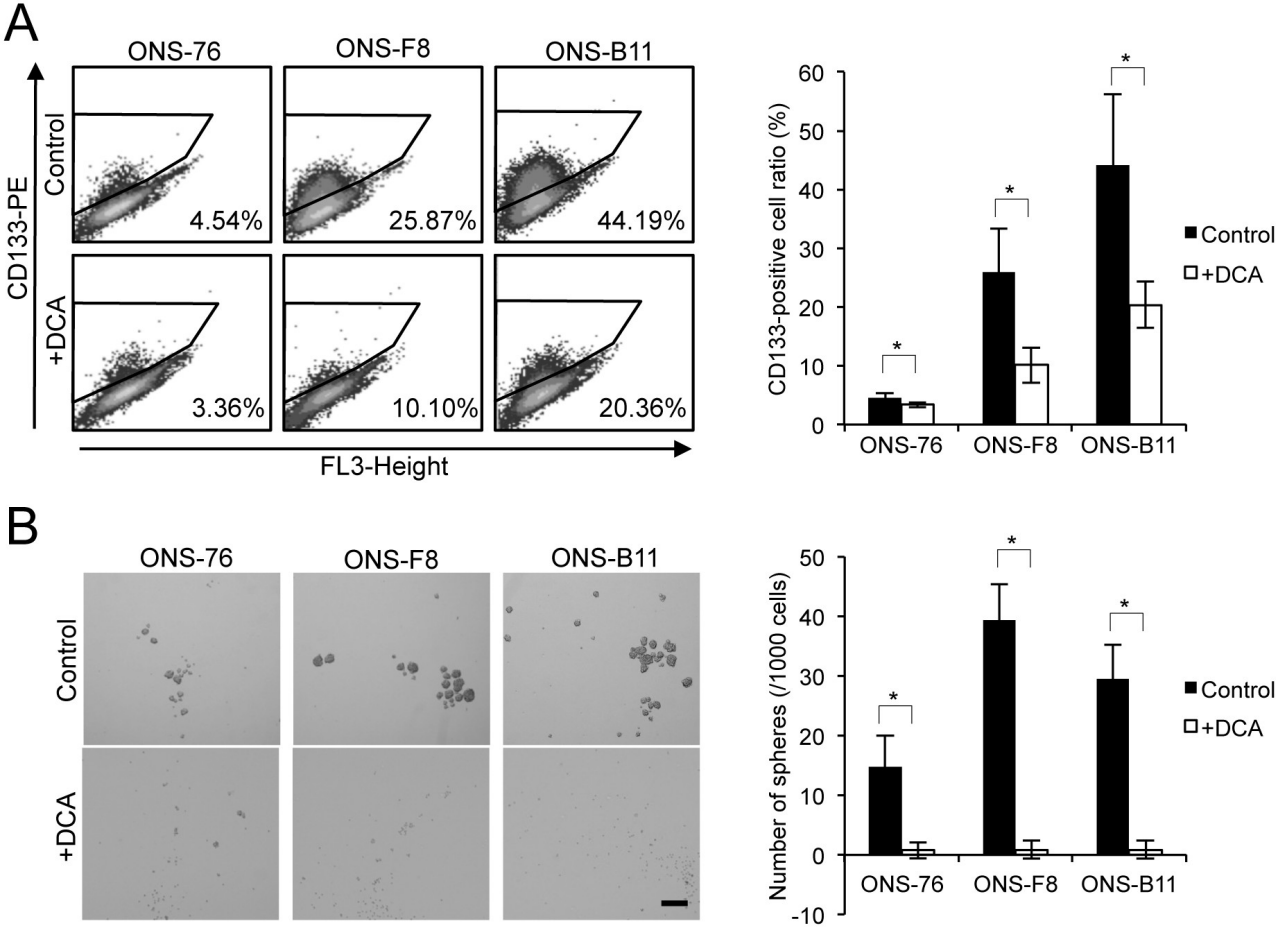
DCA suppressed cancer stem-like phenotypes *in vitro*. (A) CD133-positive cell ratio (using flow cytometry) and (B) sphere-forming ability in serum-free medium in ONS-76, -F8, and -B11 cells with and without DCA. All quantitative data are means ± S.D. *P<0.05, Welch’s t-test.

We next investigated the effect of DCA treatment on medulloblastoma cancer stem-like phenotypes by examining CD133-positivity and sphere-forming ability in control and DCA-treated cells *in vitro*. The DCA treatment groups showed significantly lower CD133-positivity and sphere-forming ratio than the control cells (Fig 5A and 5B) suggesting that DCA suppressed cancer stem-like phenotypes in rMSLCs *in vitro* and consistent with previous findings for glioma and breast CSLCs [22, 53].

### DCA increased radiosensitivity in rMSLCs through suppression of DNA repair capacity

**Fig 6:**
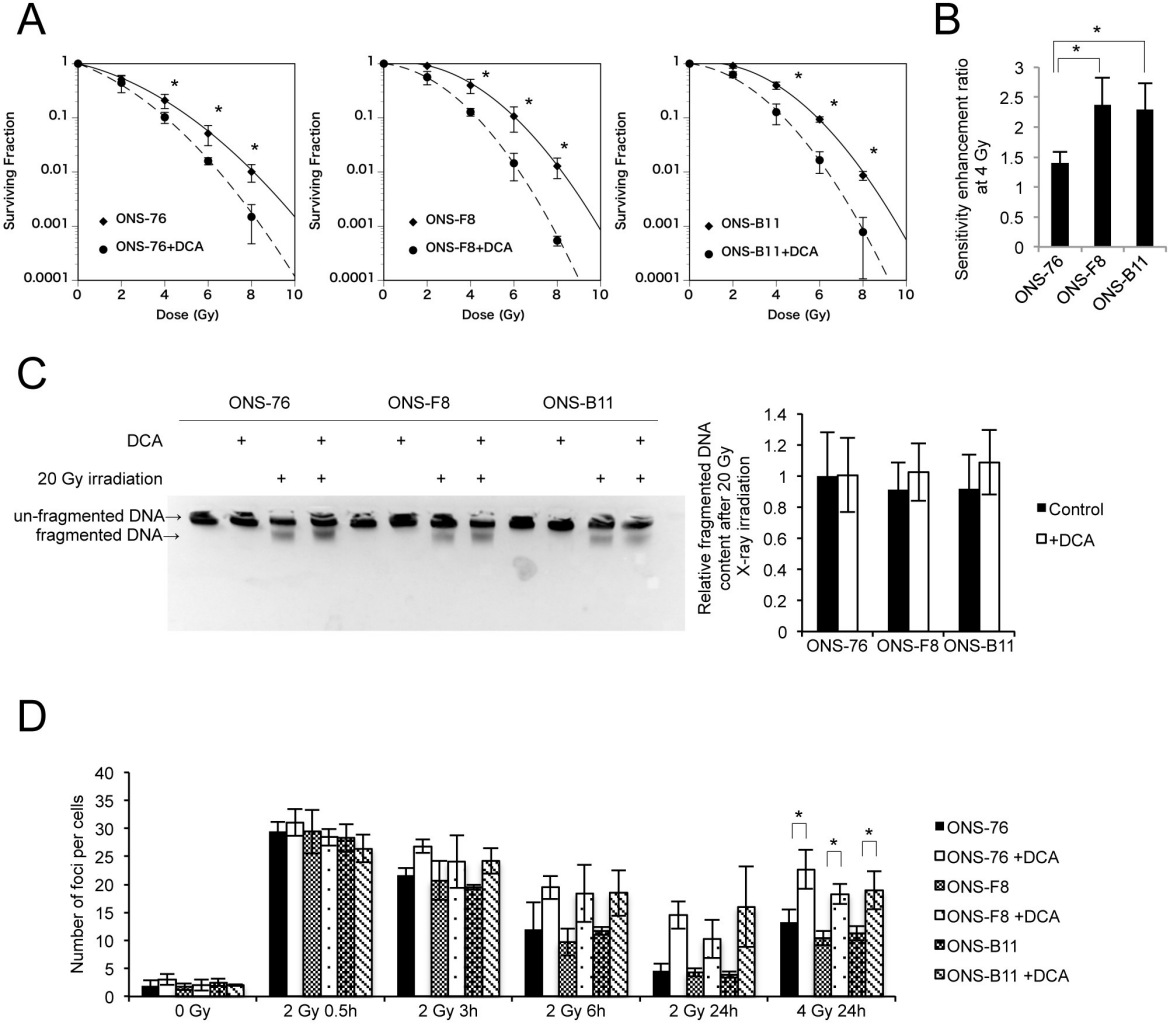
DCA enhanced radiosensitivity and suppressed DNA damage repair pathways. (A) Radiosensitivity by clonogenic survival assay, (B) sensitivity enhancement ratio at 4 Gy (calculated from result of clonogenic survival assay), (C) DNA fragmentation, (D) time response of γ-H2AX foci after irradiation with 2 or 4 Gy X-ray in ONS-76, -F8, and -B11 cells with and without DCA. All quantitative data are means ± S.D. *P<0.05, Welch’s t-test.

To investigate whether DCA treatment promotes radiosensitivity in rMSLCs we performed a clonogenic assay. As expected, DCA treatment significantly increased radiosensitivity in ONS-76, -F8, and -B11 cells after 4, 6, and 8-Gy single-dose exposure (Fig 6A). In addition, ONS-F8 and -B11 had a higher sensitivity enhancement ratio at 4 Gy than ONS-76, indicating that DCA more effectively radiosensitized ONS-F8 and -B11 (Fig 6B).

Our experimental data suggest that DCA enhances oxidative stress. We postulated that DCA-treated cells should exhibit enhanced radiation-induced DNA damage and quantified DNA damage after 20-Gy X ray irradiation with and without DCA treatment using DNA agarose gel electrophoresis. However, contrary to our initial expectations, DCA treatment did not significantly increase DNA damage in ONS-76, -F8, and -B11 cells (Fig 6C). We next investigated the kinetics of DNA double-strand break rejoining by measuring the formation and dissolution of histone γ-H2AX foci at 0.5, 3, 6, and 24 h after 2 or 4 Gy irradiation in DCA-treated and control cells. The average number of foci per cell was the same in all non-irradiated cells (Fig 6D). Both DCA-treated and control cells had a comparable increase in the average number of foci per cell at 0.5 h post irradiation (Fig 6D). However, the number of foci decreased slowly and remained high at 24 h post irradiation in the DCA-treated cells compared with the control cells, although these differences partly failed to reach statistical significance (Fig 6D). These results suggest that DCA does not enhance radiation-induced double-strand breakage but suppresses DNA repair capacity.

### Glycolysis and TCA cycle intermediate metabolites in ONS-76 and -F8 cells

To further investigate metabolic pathways (glycolysis and TCA cycle) in rMSLCs, we performed comprehensive metabolic characterization in ONS-76 and -F8 cells using CE-TOFMS and CE-QqQMS;CE-MS/MS. ONS-F8 showed a significant increase in 3-phosphoglyceric acid (3-PG), 2-phosphoglyceric acid (2-PG), and phosphoenolpyruvic acid (PEP), compared with ONS-76 (Table 1 and S4A Fig). ONS-F8 also tended to have higher intracellular levels of lactic acid and glyceraldehyde 3-phosphate and lower levels of pyruvic acid than ONS-76 (Table 1 and S4A Fig).

**Table 1:** Relative glycolysis and TCA cycle related metabolites in ONS-76 and -F8 cells with and without DCA.

	ONS-76	ONS-76 +DCA	ONS-F8	ONS-F8+DCA	Comparative Analysis*
Glucose 6-phosphate	1	13.16	0.86	10.61	2,3,4
Fructose 6-phosphate	1	12.21	0.70	8.54	2,3,4
Fructose 1,6-diphosphate	1	13.83	1.15	14.17	1,2,3
Glyceraldehyde 3-phosphate	N.D.	>43.66	>6.56	>50.24	
Dihydroxyacetone phosphate	N.D.	>92.31	N.D.	>101.7	
2,3-Diphosphoglyceric acid	1	1.69	0.79	1.59	1,2,3
3-Phosphoglyceric acid	1	0.94	1.46	0.85	1,3
2-Phosphoglyceric acid	1	1.01	1.41	1.01	1
Phosphoenolpyruvic acid	1	N.D.	5.20	N.D.	1
Pyruvic acid	1	2.60	0.75	1.89	2
Intracellular lactic acid	1	14.11	1.33	13.88	2,3
Acetyl CoA	1	22.86	0.78	21.71	2
Citric acid	1	0.99	0.87	0.80	1
*cis*-Aconitic acid	1	0.94	0.96	0.76	3
Isocitric acid	1	0.60	1.34	0.63	1,3
2-Oxoglutaric acid	1	N.D.	2.70	N.D.	
Succinic acid	1	0.59	0.77	0.49	1,2,3
Fumaric acid	1	2.77	2.29	2.37	
Malic acid	1	0.68	1.09	0.73	
ATP	1	0.76	0.77	0.60	1,2,3,4
ADP	1	1.31	0.87	1.07	
AMP	1	1.23	0.92	0.92	2,4
Total Adenylate	1	0.78	0.77	0.62	1,2,3,4
ATP/ADP	1	0.59	0.91	0.56	2,3
Adenylate Energy Charge	1	0.99	1	0.98	2,3
NADH	1	1.32	0.85	1.07	1,2,3,4
NAD^+^	1	0.97	0.80	0.71	1,3,4
NADH/ NAD^+^	1	1.36	1.06	1.52	2,3
Alanin	1	0.71	0.85	0.43	1,2,3,4
Arginine	1	1.29	0.83	1.08	1,2,3,4
Asparagine	1	7.08	0.80	5.12	1,2,3,4
Aspartic Acid	1	2.64	0.65	1.85	1,2,3,4
Cysteine	1	0.30	0.75	0.34	1,2,3
Glutamine	1	64.96	1.19	58.65	2,3
Glutamic Acid	1	1.71	1.01	1.36	2,3,4
Glycine	1	2.37	0.28	1.46	1,2,3,4
Histidine	1	2.35	0.90	2.21	1,2,3,4
Isoleucine	1	3.22	0.90	2.89	1,2,3,4
Leucine	1	3.53	0.90	3.26	1,2,3
Lysine	1	1.68	0.83	1.40	1,2,3,4
Methionine	1	4.96	0.94	5.06	2,3
Phenylalanine	1	2.52	0.92	2.37	2,3
Proline	1	1.95	0.75	1.57	1,2,3,4
Serine	1	1.75	0.31	0.84	1,2,3,4
Threonine	1	1.91	0.91	1.62	1,2,3,4
Tryptophan	1	2.75	0.89	2.69	1,2,3
Tyrosine	1	2.50	0.92	2.34	2,3
Valine	1	2.63	0.93	2.37	1,2,3

*: Comparative analysis was performed by Welch’s t test. A P value less than 0.05 was considered statistically significant.

1: significant difference in ONS-76 vs. ONS-F8

2: significant difference in ONS-76 vs. ONS-76+DCA

3: significant difference in ONS-F8 vs. ONS-F8+DCA

4: significant difference in ONS-76+DCA vs. ONS-F8+DCA

With respect to TCA cycle intermediates, ONS-F8 showed significantly increased levels of isocitric acid and decreased citric acid and succinic acid compared with ONS-76 (Table 1 and S4B Fig). ONS-F8 also tended to have higher 2-oxoglutaric acid (2-OG) levels and lower acetyl CoA levels than ONS-76 (Table 1 and S4B Fig). Interestingly, ONS-F8 also showed significantly decreased levels of oxidized nicotinamide adenine dinucleotide (NAD^+^) and reduced nicotinamide adenine dinucleotide (NADH) compared with ONS-76 (Table 1 and S4B Fig). These results, together with our OCR data, indicate that ONS-F8 had both reduced electron transport chain and oxidative phosphorylation activities compared with ONS-76. Furthermore, ONS-F8 showed significantly low ATP and total adenylate levels, but maintained the same ATP/ADP ratio and adenylate energy charge (AEC) [54] as ONS-76 (Table 1 and S4C Fig), indicating that ONS-F8 exists in a low-energy or quiescent metabolic state, but does not lack ATP.

### DCA induces alteration of glucose catabolic pathway at multiple steps and results in cellular energy depletion

Mass spectrometry analyses showed that DCA-treated cells exhibited increased glucose 6-phosphate (G6P), fructose 6-phosphate (F6P), fructose 1,6-diphosphate (F1,6P), dihydroxyacetone phosphate (DHAP), glyceraldehyde 3-phosphate (GAP), 2,3-diphosphoglyceric acid, and NADH/NAD^+^ ratio, and depleted PEP, compared with control groups (Table 1 and S4A and S4B Fig). These results suggest that a high NADH/NAD^+^ ratio induced accumulation of early metabolites in glycolysis [55]. Furthermore, DCA increased accumulation of pyruvic acid, acetyl CoA, and intracellular lactic, and decreased extracellular lactate production (Table 1 and S4A and S4B Fig). More importantly, DCA treatment did not change levels of citric acid but increased those of acetyl CoA, suggesting that the conversion of acetyl CoA to citric acid was suppressed (Table 1 and S4B Fig).

Interestingly, DCA decreased levels of isocitric acid, succinic acid, malic acid, and 2-OG, although these differences partly failed to reach statistical significance, and no TCA cycle intermediates showed increased levels after DCA treatment (Table 1 and S4B Fig). We also found accumulation of 18 amino acids in DCA-treated cells while only two (alanine and cysteine) showed decreased levels (Table 1 and S4D Fig). Furthermore, DCA treatment groups had significantly lower ATP levels, ATP/ADP ratio, and AEC than control groups (Table 1 and S4C Fig). Our results indicate that DCA induced energy depletion and alteration of mitochondria ROS metabolism through modulation of glycolysis, TCA cycle, and amino acid metabolism.

## Discussion

In the present study we investigated the contribution and importance of energy metabolic properties to radioresistance and maintenance of stem cell-like characteristics of rMSLCs generated after irradiation. Our results indicated suppressed mitochondrial respiration (Fig 2) in rMSLCs. In particular, low endogenous mitochondria ROS production,sustained lower levels of ROS in rMSLCs (Figs 1 and 2). DCA treatment led to prominent perturbations of multiple energy production processes, such as glucose metabolism restriction Table 1 and S4A Fig), mitochondria morphological changes (Fig 3F and 3G), and increased endogenous mitochondria ROS production (Fig 3B), resulting in oxidative stress (Fig 3A and 3C), failure of DNA repair after irradiation (Fig 5), and loss of cancer stem cell-like phenotypes (Fig 4).

**Fig 7:**
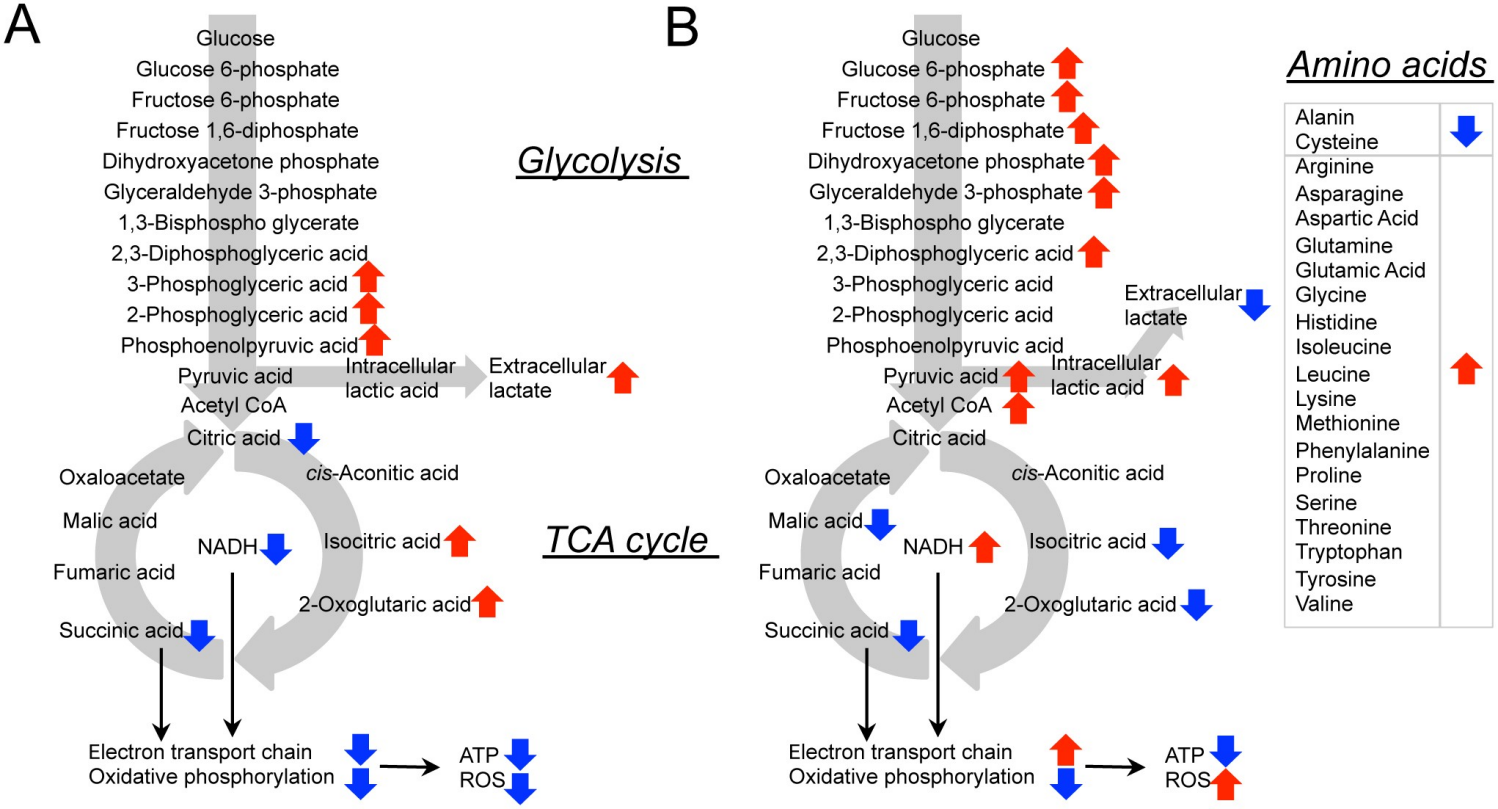
ONS-76, -F8 and DCA-treated cells exhibit different metabolic profiles. Schematic diagram of the metabolic pathway. The red and blue arrows represent increased and decreased metabolite concentrations in ONS-F8 compared with ONS-76 (A), and in DCA-treated cells compared with non-DCA-treated cells (B).

Previous reports have demonstrated that CSLCs have higher radioresistance than non-CSLCs as a result of increased DNA repair capability and antioxidative capacity [14, 15, 56], suggesting that these properties could be a potential target of CSLC-based therapy. Metabolic pathways, such as glycolysis, the TCA cycle, and the pentose phosphate pathway, are not only key regulators of ROS production and antioxidant biosynthesis, but also play a pivotal role in cancer cell radioresistance [57, 58]. Thus, to understand CSLC radioresistance these metabolic pathways should be closely investigated. Vlashi et al. examined metabolic differences between glioma stem cell-enriched populations and differentiated glioma cells, concluding that glioma stem cell-enriched cells are less glycolytic than differentiated glioma cells [25]. Consistent with this study, Lagadinou et al. revealed that low-ROS leukemia stem cells are highly reliant on oxidative phosphorylation [23]. Conversely, Feng et al. showed that breast tumor initiating cells preferentially perform glycolysis over oxidative phosphorylation compared with non-tumorigenic cancer cells [22]. However, no previous papers have revealed the status of metabolic properties, cellular redox state, and radioresistance in medulloblastoma CSLCs. We previously established rMSLCs (ONS-F8 and -B11) from a medulloblastoma cell line (ONS-76). These cells can be maintained in the same culture conditions and can be analyzed in the same culture period as the parental medulloblastoma cell line. Assessment of mitochondria superoxide production, OCR value (Fig 2C), and intermediates in TCA metabolism (Table 1, S4B Fig and Fig 7A) for rMSLCs and the parental cell line suggested that rMSLCs have lower mitochondria respiration. rMSLCs and the parental cell line showed the same level of glucose uptake (Fig 4D). Moreover, PYK activity (Fig 4C), lactate production (Fig 4B), and concentrations of PEP, pyruvic acid, intracellular lactic acid, acetyl CoA, and citric acid (Table 1, S4A and S4B Fig and Fig 7A) suggested that rMSLCs had a high rate of conversion of pyruvic acid to lactic acid and a low rate of conversion of pyruvic acid to acetyl CoA compared with the parental cell line. Intriguingly, compared with ONS-76, mitochondrial mass and potential were lower in ONS-F8 but the same in ONS-B11 (Fig 2A and 2B). These results may suggest that the **diminution** of mitochondria respiration is supported by different molecular mechanisms in ONS-F8 and ONS-B11, and glycolytic activity and oxidative phosphorylation do no show a direct relationship with CD133 positivity. Radiation or long-term use of 3-aminobenzamide (a PARP inhibiter) induced transformation of glioma stem-like cells or osteosarcoma cells into highly glycolysis-dependent CSLCs [59–61], suggesting that therapeutic strategies could combine standard radiation or chemotherapy and inhibition of glycolysis.

It has been reported that several CSLCs, including breast, hepatic, and colon CSLCs, display low intracellular ROS levels through high antioxidant capacity compared with non-CSLCs [15, 16, 47]. Although our data showed that rMSLCs possess low intracellular ROS levels and low nuclear oxidative stress levels, no significant differences in GSH levels were observed between rMSLCs and the parental line (Fig 1A, 1C and 1D). Our data suggest that endogenous mitochondria superoxide production was suppressed in rMSLCs through low oxidative phosphorylation activity. Pluripotent stem cells and hematopoietic stem cells (HSCs) also maintain low levels of ROS through suppressed mitochondria ROS production as a result of reduced reliance upon oxidative phosphorylation and limited mitochondria biogenesis [62–65]. Pdk, a metabolic enzyme that is suppressed during mitochondrial respiration through inhibition of Pdh, shows increased activity in induced pluripotent stem (iPS) cells and HSCs [20, 65]. Loss of Pdk attenuates HSC quiescence, glycolysis, and transplantation capacity [65]. These results are highly concordant with our data showing that treatment with the Pdk inhibitor DCA induced increased mitochondria mass and superoxide production (Fig 3B and 3D), and attenuated CD133-positivity and sphere formation (Fig 5). This suggests that Pdk-mediated antagonism of mitochondria metabolism and a low ROS state are favored for maintenance of stem cell properties in both normal stem cells and rMSLCs. Indeed, Tsatmali et al. and Buggisch et al. have demonstrated that NADPH oxidase and subsequent ROS accumulation and signaling play an important role in ES cell differentiation [66, 67]. This issue should be explored in more detail.

We also demonstrated that DCA treatment increased radiosensitivity in rMSLCs though suppressed DNA repair capacity but did not enhance the initial radiation-induced ROS-mediated DNA damage (Fig 6). Our results suggest three potential mechanisms by which DCA might suppress DNA repair. First, DCA treatment induced oxidization in the nucleus (Fig 3C) and these highly oxidized conditions might hinder DNA repair after irradiation. It has been reported that enhancement of oxidative stress, such as by H_2_O_2_ treatment or zinc deficiency, induces dysfunction of DNA repair proteins, and hence compromises DNA repair [68, 69]. Second, DCA treatment induced high acetyl CoA levels that can complicate DNA repair (Table 1 and S4B Fig). It has been shown that DCA increases histone acetylation associated with acetyl CoA accumulation, and that inhibition of histone deacetylase enhances radiosensitivity through prolongation of γ-H2AX foci [28, 70]. Third, DCA treatment induced low ATP levels and an energy depletion state (Table 1 and S4C Fig) might affect DNA repair after irradiation, which is known to require ATP [71, 72]. Which mechanism contributes most to enhancement of radiosensitivity in rMSLCs is unclear and requires further investigation.

Moreover, our results showed that DCA treatment modified multiple energy production processes (Table 1, S4 Fig and Fig 7B). First, DCA treatment increased mitochondrial mass (Fig 3D) and membrane potential (Fig 3E), and changed mitochondria shape to an elongated form (Fig 3F and 3G). It is possible that these mitochondrial changes led to increased superoxide production and high levels of oxidative stress (Fig 3A–3C). Further, DCA treatment induced low ATP levels and an energy depletion state (Table 1 and S4C Fig). It has been demonstrated that ROS production increases after blocking OxPhos [73, 74]. Our results suggest that DCA treatment reduces oxidative phosphorylation through ROS accumulation. Second, DCA treatment led to a decrease in several TCA cycle intermediates, including isocitric acid, succinic acid, malic acid, and 2-OG (Table 1, S4B Fig). Conversely, levels of 18 amino acids, all of which could be catabolized to pyruvic acid or TCA cycle intermediates, were increased (Table 1, S4D Fig) implying that DCA induced dampening of the TCA cycle flux through inhibition of amino acid degradation. Instead, DCA-enhanced pyruvic acid-acetyl CoA conversion might be responsible for maintenance of the NADH supply (Table 1, S4B Fig). Additionally, we showed that accumulated high levels of acetyl CoA did not elevate citric acid levels (Table 1, S4B Fig), consistent with a previous study in murine hearts [28]. Third, DCA treatment induced accumulation of early-stage glycolysis intermediates (before 3-PG) (Table 1, S4A Fig), suggesting inhibition of GAPDH that catalyzes conversion of glyceraldehyde 3-phosphate into 1,3-bisphosphoglycerate, with a high NADH/NAD^+^ ratio (Table 1, S4A Fig) [55]. Fourth, DCA-treated cells showed decreased alanine levels and increased pyruvic acid levels, suggesting that DCA also promoted conversion of alanine into pyruvic acid (Table 1, S4D Fig). Last, DCA caused accumulation of intracellular lactic acid and a decrease in extracellular lactate production (Fig 4B, Table 1 and S4D Fig). It is possible that DCA enhanced extracellular lactate uptake or suppressed lactate excretion. Taken together, our data suggest that DCA treatment did not simply activate the TCA cycle and oxidative phosphorylation, but instead led to multiple metabolic pathway alterations in rMSLCs. DCA is considered a potentially effective drug for cancer cells and CSLCs at the experimental level and several clinical trials are currently underway. Our data provide a more comprehensive understanding of the effect of DCA treatment on rMSLCs, which represents a milestone in investigations of the molecular association of stem cell-like phenotypes and metabolic pathways in medulloblastoma CSLCs and the development of novel drugs targeting medulloblastoma CSLCs.

It has been pointed out that cancer cell metabolism is different between in vivo tumors and in vitro cell lines. In addition, several other studies indicated that glycolytic ATP contribution differs greatly among cancers (accounting for 5–50% of the cellular ATP) [75, 76]. Oncogene aberrations and/or the tumor microenvironment are major causes of metabolic changes in tumor cells [77, 78] and it is important to consider these backgrounds when analyzing metabolic data. Furthermore, mitochondria in tumor cells consume not only pyruvate, but also glutamine, free fatty acids, ketone bodies, and proline [79, 80]. Although the lack of data on these metabolites is a limitation of this study, it is outside the scope of this article. Future investigations are needed to examine glutamine, free fatty acids, ketone bodies, and proline metabolism, and whether these metabolic pathways are associated with radioresistance and stem cell phenotype.

## Conclusion

We showed that rMSLCs had low ROS levels resulting from suppression of mitochondria oxidative phosphorylation. DCA treatment suppressed cancer stem cell-like phenotypes and increased intracellular ROS levels and radiosensitivity by suppressing glycolysis and inducing mitochondrial aberrations. Combined therapy with metabolic targeted drugs and radiation might be effective for eradication of medulloblastoma CSLCs.

## Supporting information

S1 Fig Establishment of radioresistant medulloblastoma stem cell-like clones. (A) Summary of cancer stem cell-like cell phenotypes *in vitro* (reprinted from our previous report). (B) Tumorigenicity of ONS-76, -F8, and -B11 cells in NOD/SCID mice. Cells were injected subcutaneously into male NOD/SCID mice and tumor formation was observed for 16 weeks after injection. Tumorigenic cell frequencies were calculated using the formula available on the WEHI ELDA website.
(PDF)

S2 Fig Diminution of OCR in ONS-F8 and -B11 cells. OCR (using ESR oximetry) in ONS-76, -F8 and -B11 cells. All quantitative data are means ± S.D. *P<0.05, Welch’s t-test.
(PDF)

S3 Fig Cellular toxicity of DCA treatment in ONS-76, ONS-F8, and ONS-B11 cells. (A) Ratio of PI-positive (dead) cells by flow cytometry, (B) Cell survival ratio after DCA treatment by clonogenic survival assay. Cells were treated with 50 mM DCA for 48 h. All quantitative data are means ± S.D. *P<0.05, Welch’s t-test, n.s., non-significant.
(PDF)

S4 Fig Metabolome analysis in ONS-76 and -F8 cells with and without DCA. (A) Glycolysis, (B) TCA cycle, NADH, and NAD^+^, (C) ATP, ADP, and AMT, and (D) amino acids in ONS-76, -F8 and -B11 cells. All quantitative data are means ± S.D. *P<0.05, Welch’s t-test.
(PDF)

S5 Fig Concentration of phosphoenolpyruvic acid, pyruvic acid, intracellular lactic acid, acetyl CoA, and citric acid in ONS-76 and -F8 cells. All quantitative data are means ± S.D. *P<0.05, Welch’s t-test.
(PDF)
