# Supplementary material for: Metabolic analysis of radioresistant medulloblastoma stem-like clones and potential therapeutic targets
Source: PLoS One. 2017 Apr 20;12(4):e0176162. doi: 10.1371/journal.pone.0176162 (PMC5398704; doi:10.1371/journal.pone.0176162)
Supplement: S1 Fig — (A) Summary of cancer stem cell-like cell phenotypes in vitro (reprinted from our previous report). (B) Tumorigenicity of ONS-76, -F8, and -B11 cells in NOD/SCID mice. Cells were injected subcutaneously into male NOD/SCID mice and tumor formation was observed for 16 weeks after injection. Tumorigenic cell frequencies were calculated using the formula available on the WEHI ELDA website. (PDF) [file pone.0176162.s001.pdf]

S1 Fig

A

In vitro stemness characterization of ONS-76, F8, B11 and F11

	CD133 ratio (%)	Sphere forming cell frequency	Side population ratio (%)	Radioresistance (SF2)
ONS-76 (parental cell)	3.77	1/23.2	0.14	0.64
ONS-F8	25.30*	1/10.8*	0.26*	0.82*
ONS-B11	43.91*	1/10.3*	0.33*	0.90*
ONS-F11	14.19*	1/13.2*	0.24*	0.81*

Sphere forming cell frequency were calculated by limiting dilution assay.
Values of SF2 indicate surviving fractions at dose of 2 Gy.
*indicated significant differences (vs. ONS-76, Student-t test, $P < 0.05$)
These data have been published in JRR. 2013; 54(1)

B

Tumorigenicity of ONS-76, ONS-F8 and ONS-B11 cells in NOD/SCID mice

	Cell Numbers for Injection				
	1×10^6	1×10^4	1×10^3	1×10^2	Tumorigenic cell frequency (P value, vs. ONS-76)
ONS-76	6/6	4/6	4/6	0/6	1/4747
ONS-F8	6/6	5/6	5/6	5/6	1/1351 ($p < 0.05$)
ONS-B11	6/6	5/6	5/6	4/6	1/1508 ($p < 0.05$)

Note: The cells were injected into the subcutaneous of male NOD/SCID mice.
Tumor formation was observed for 16 weeks after injection.
Tumorigenic cell frequency were calculated by the formula available on the WEHI ELDA website (http://bioinf.wehi.edu.au/software/elda/).

S1 Fig. Establishment of radioresistant medulloblastoma stem cell-like clones. (A) Summary of cancer stem cell-like cell phenotypes in vitro (reprinted from our previous report). (B) Tumorigenicity of ONS-76, -F8, and -B11 cells in NOD/SCID mice. Cells were injected subcutaneously into male NOD/SCID mice and tumor formation was observed for 16 weeks after injection. Tumorigenic cell frequencies were calculated using the formula available on the WEHI ELDA website.
