# Supplementary material for: Metabolic analysis of radioresistant medulloblastoma stem-like clones and potential therapeutic targets
Source: PLoS One. 2017 Apr 20;12(4):e0176162. doi: 10.1371/journal.pone.0176162 (PMC5398704; doi:10.1371/journal.pone.0176162)
Supplement: S2 Fig — OCR (using ESR oximetry) in ONS-76, -F8 and -B11 cells. All quantitative data are means ± S.D. *P<0.05, Welch’s t-test. (PDF) [file pone.0176162.s002.pdf]

S2 Fig

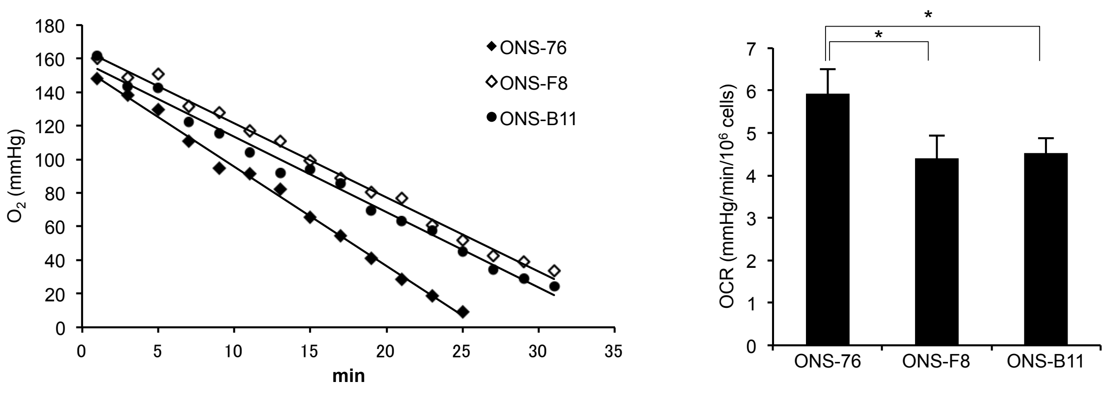

S2 Fig. Diminution of OCR in ONS-F8 and -B11 cells. OCR (using ESR oximetry) in ONS-76, -F8 and -B11 cells. All quantitative data are means ± S.D. *P<0.05, Welch's t-test.
