# Supplementary material for: Metabolic analysis of radioresistant medulloblastoma stem-like clones and potential therapeutic targets
Source: PLoS One. 2017 Apr 20;12(4):e0176162. doi: 10.1371/journal.pone.0176162 (PMC5398704; doi:10.1371/journal.pone.0176162)
Supplement: S3 Fig — (A) Ratio of PI-positive (dead) cells by flow cytometry, (B) Cell survival ratio after DCA treatment by clonogenic survival assay. Cells were treated with 50 mM DCA for 48 h. All quantitative data are means ± S.D. *P<0.05, Welch’s t-test, n.s., non-significant. (PDF) [file pone.0176162.s003.pdf]

S3 Fig

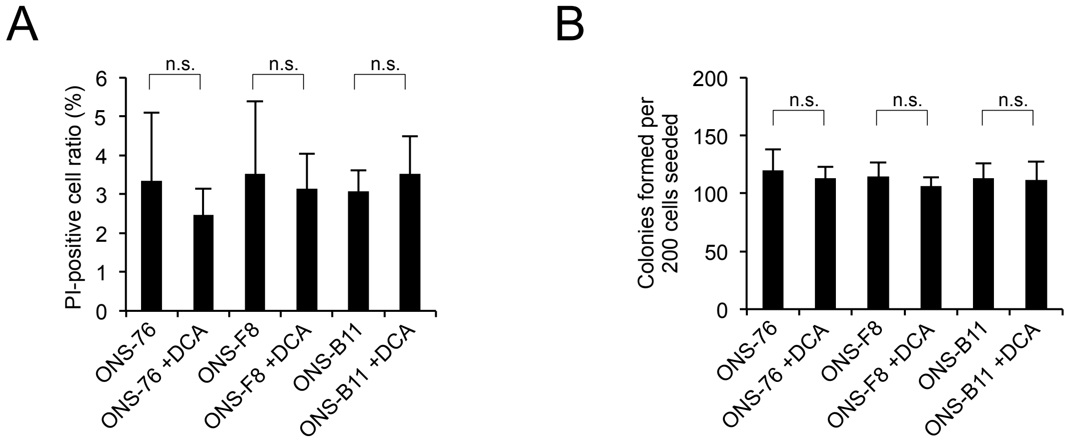

S3 Fig. Cellular toxicity of DCA treatment in ONS-76, ONS-F8, and ONS-B11 cells. (A) Ratio of PI-positive (dead) cells by flow cytometry, (B) Cell survival ratio after DCA treatment by clonogenic survival assay. Cells were treated with 50 mM DCA for 48 h. All quantitative data are means ± S.D. *P<0.05, Welch's t-test, n.s., non-significant.
