# Supplementary material for: Metabolic analysis of radioresistant medulloblastoma stem-like clones and potential therapeutic targets
Source: PLoS One. 2017 Apr 20;12(4):e0176162. doi: 10.1371/journal.pone.0176162 (PMC5398704; doi:10.1371/journal.pone.0176162)
Supplement: S4 Fig — (A) Glycolysis, (B) TCA cycle, NADH, and NAD+, (C) ATP, ADP, and AMT, and (D) amino acids in ONS-76, -F8 and -B11 cells. All quantitative data are means ± S.D. *P<0.05, Welch’s t-test. (PDF) [file pone.0176162.s004.pdf]

S4 Fig A

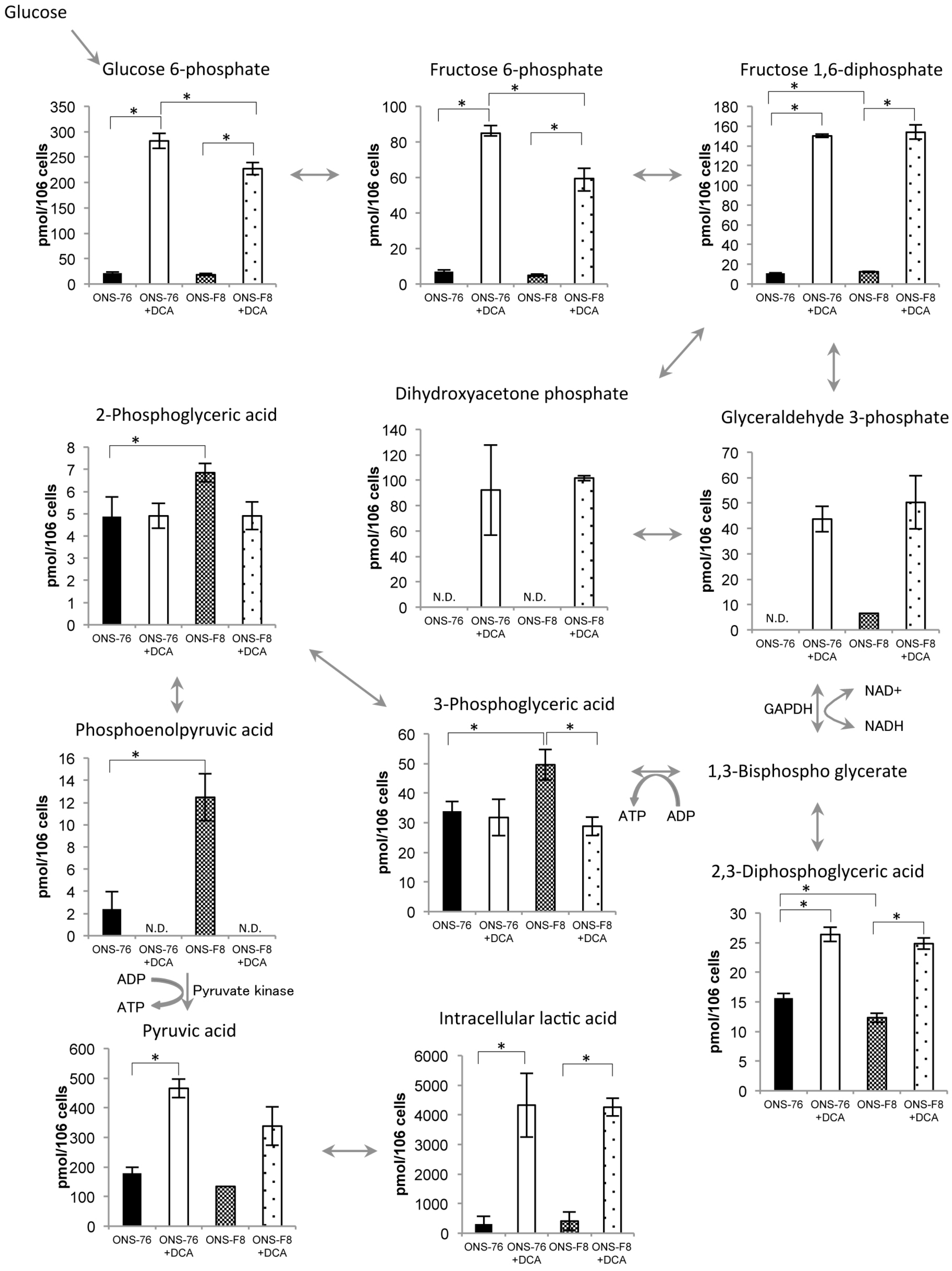
Glucose
Glucose 6-phosphate
Fructose 6-phosphate
Fructose 1,6-diphosphate
Dihydroxyacetone phosphate
Glyceraldehyde 3-phosphate
2-Phosphoglyceric acid
GAPDH
NAD+
NADH
1,3-Bisphospho glycerate
ATP
ADP
3-Phosphoglyceric acid
2,3-Diphosphoglyceric acid
Phosphoenolpyruvic acid
ADP
ATP
Pyruvate kinase
Pyruvic acid
Intracellular lactic acid
pmol/106 cells
ONS-76
ONS-76 +DCA
ONS-F8
ONS-F8 +DCA
N.D.

S4 Fig B

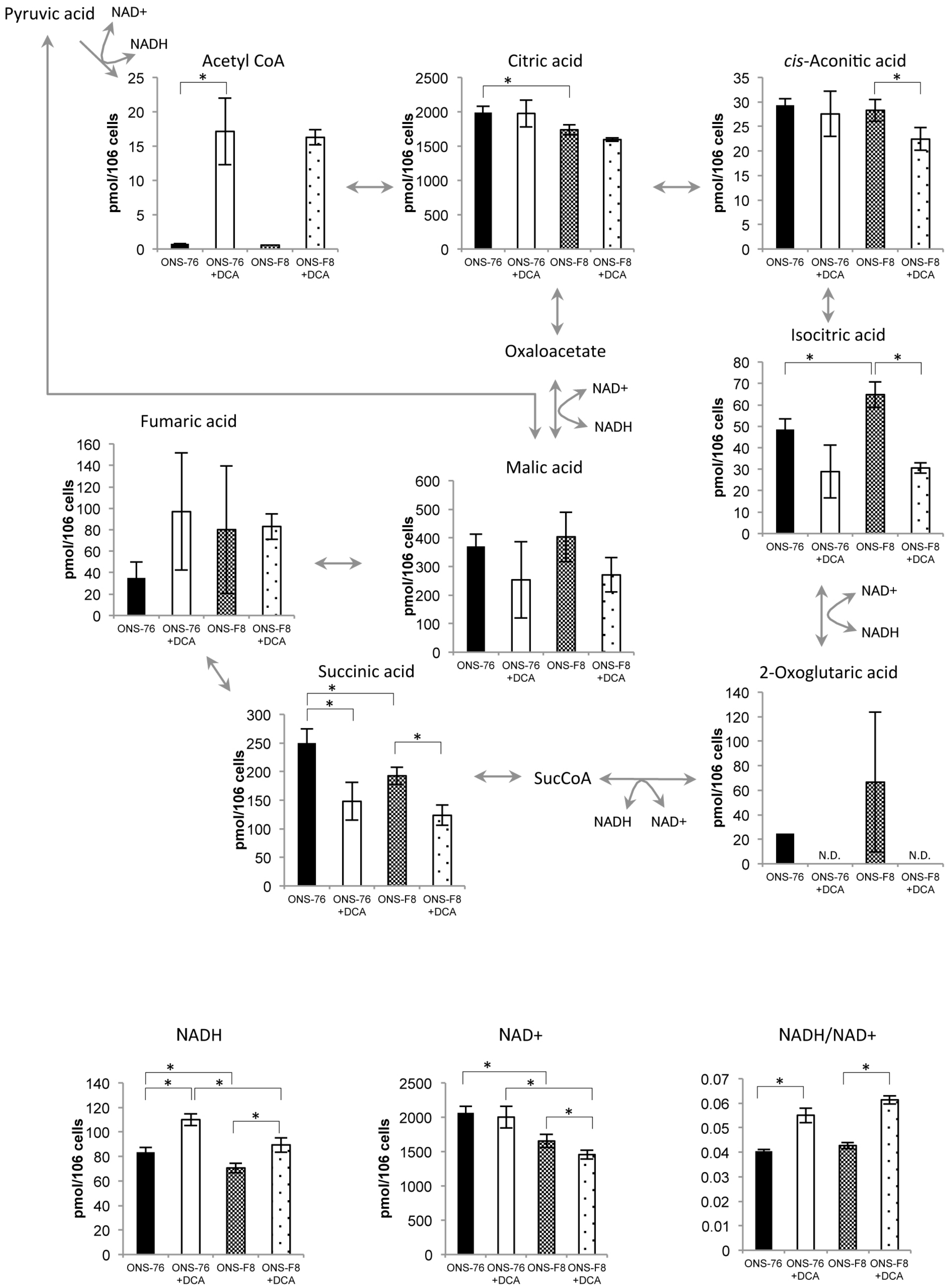
Pyruvic acid
NAD+
NADH
Acetyl CoA
Citric acid
cis-Aconitic acid
Isocitric acid
Oxaloacetate
Malic acid
Fumaric acid
Succinic acid
SucCoA
2-Oxoglutaric acid
NADH
NAD+
NADH/NAD+
pmol/106 cells
ONS-76
ONS-76 +DCA
ONS-F8
ONS-F8 +DCA
N.D.
*

S4 Fig C

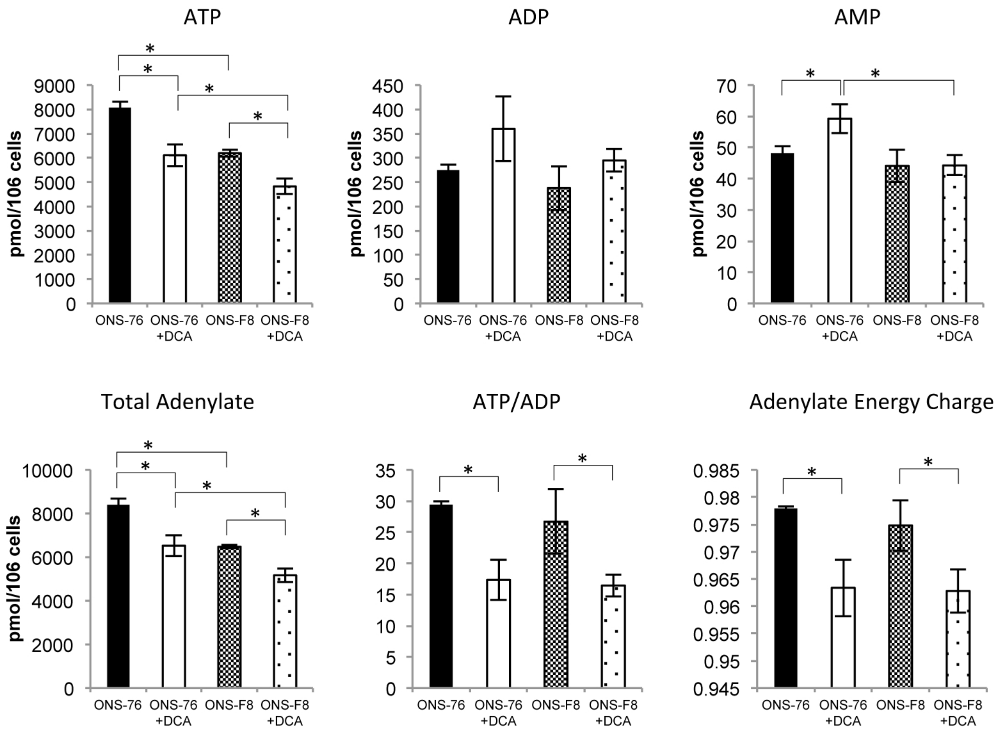
ATP
ADP
AMP
Total Adenylate
ATP/ADP
Adenylate Energy Charge
pmol/106 cells
ONS-76
ONS-76 +DCA
ONS-F8
ONS-F8 +DCA
*

S4 Fig D

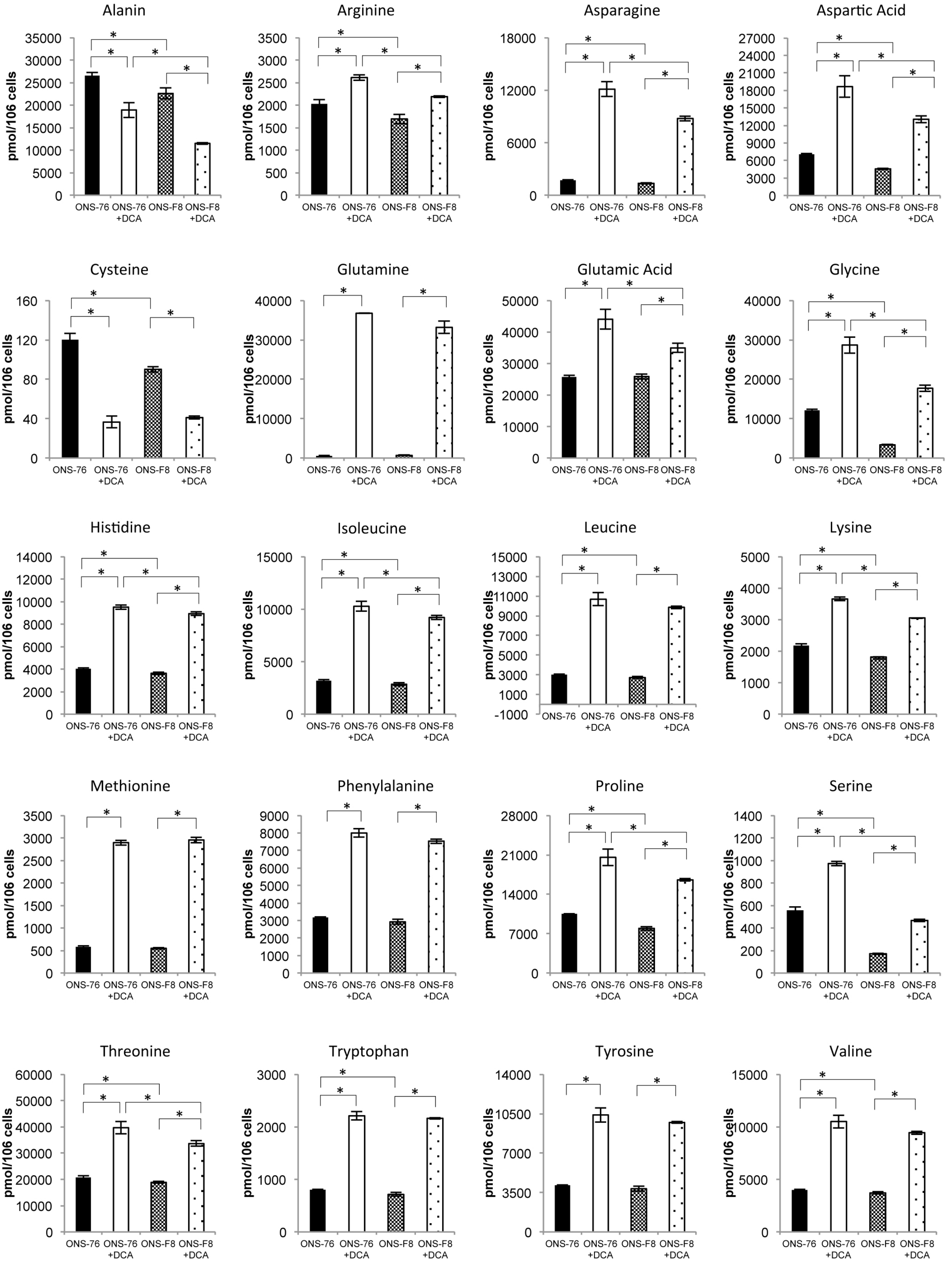
Alanin
pmol/106 cells
0 5000 10000 15000 20000 25000 30000 35000
Arginine
pmol/106 cells
0 500 1000 1500 2000 2500 3000 3500
Asparagine
pmol/106 cells
0 6000 12000 18000
Aspartic Acid
pmol/106 cells
0 3000 6000 9000 12000 15000 18000 21000 24000 27000
Cysteine
pmol/106 cells
0 40 80 120 160
Glutamine
pmol/106 cells
0 10000 20000 30000 40000
Glutamic Acid
pmol/106 cells
0 10000 20000 30000 40000 50000
Glycine
pmol/106 cells
0 10000 20000 30000 40000
Histidine
pmol/106 cells
0 2000 4000 6000 8000 10000 12000 14000
Isoleucine
pmol/106 cells
0 5000 10000 15000
Leucine
pmol/106 cells
-1000 1000 3000 5000 7000 9000 11000 13000 15000
Lysine
pmol/106 cells
0 1000 2000 3000 4000 5000
Methionine
pmol/106 cells
0 500 1000 1500 2000 2500 3000 3500
Phenylalanine
pmol/106 cells
0 1000 2000 3000 4000 5000 6000 7000 8000 9000
Proline
pmol/106 cells
0 7000 14000 21000 28000
Serine
pmol/106 cells
0 200 400 600 800 1000 1200 1400
Threonine
pmol/106 cells
0 10000 20000 30000 40000 50000 60000
Tryptophan
pmol/106 cells
0 1000 2000 3000
Tyrosine
pmol/106 cells
0 3500 7000 10500 14000
Valine
pmol/106 cells
0 5000 10000 15000
ONS-76
ONS-76 +DCA
ONS-F8
ONS-F8 +DCA

S4 Fig. Metabolome analysis in ONS-76 and -F8 cells with and without DCA. (A) Glycolysis, (B) TCA cycle, NADH, and NAD+, (C) ATP, ADP, and AMT, and (D) amino acids in ONS-76, -F8 and -B11 cells. All quantitative data are means ± S.D. *P<0.05, Welch's t-test.
