# Supplementary material for: Metabolic analysis of radioresistant medulloblastoma stem-like clones and potential therapeutic targets
Source: PLoS One. 2017 Apr 20;12(4):e0176162. doi: 10.1371/journal.pone.0176162 (PMC5398704; doi:10.1371/journal.pone.0176162)
Supplement: S5 Fig — All quantitative data are means ± S.D. *P<0.05, Welch’s t-test. (PDF) [file pone.0176162.s005.pdf]

S5 Fig

	Concentration (pmol/10^6 cells)						ONS-F8/ ONS-76	P value (Welch's t test)
	ONS−76			ONS−F8				
	trial 1	trial 2	trial 3	trial 1	trial 2	trial 3		
Phosphoenolpyruvic acid (PEP)	1.6	4.2	1.4	11.6	10.9	14.9	5.2	0.003
Pyruvic acid	202	165	169	N.D.	N.D.	134	0.75	N.A.
Intracellular lactic acid	525	371	24	762	174	290	1.3	0.7
Acetyl CoA	0.78	0.77	0.70	N.D.	N.D.	0.58	0.78	N.A.
Citric acid	2067	2017	1881	1819	1710	1681	0.87	0.03

S5 Fig. Concentration of phosphoenolpyruvic acid, pyruvic acid, intracellular lactic acid, acetyl CoA, and citric acid in ONS-76 and -F8 cells. All quantitative data are means ± S.D. *$P<0.05$, Welch's t-test.
